# Intracranial hypotension after trauma

**DOI:** 10.1186/2193-1801-3-153

**Published:** 2014-03-21

**Authors:** Asita S Sarrafzadeh, Stephanie A Hopf, Oliver P Gautschi, Ana-Paula Narata, Karl Schaller

**Affiliations:** Division of Neurosurgery, Department of Clinical Neurosciences, Faculty of Medicine, Geneva University Medical Center, Geneva, Switzerland; Department of Interventional and Diagnostic Neuroradiology, University Hospital of Geneva, Geneva, Switzerland

**Keywords:** TBI, Intracranial hypotension, Chronic subdural hematoma

## Abstract

**Introduction:**

Intracranial hypotension (IH) occurs typically spontaneous and is a potentially life-threatening condition characterized by symptoms varying from postural headache to coma, with classical magnetic resonance imaging (MRI) findings.

**Case description:**

We report two cases of clinically relevant trauma-related IH and review of the literature.

One patient with a cerebral trauma presented unilateral mydriasis and coma resolved by the Trendelenburg position (-20°) as urgency intervention. In the second patient, IH was caused by a lesion of the brachial plexus after a motor vehicle accident.

**Discussion and conclusion:**

A history of mild or moderate trauma in association with prolonged postural or permanent headache may indicate IH. Posttraumatic IH is rare, nevertheless life-threatening in case of misdiagnosis. Intracranial hypotension in a trauma context is rarely described and difficult to diagnose. The change from tipical supine 30° to Trendelenburg position (0–20°) can be a life-saving manoeuver in these patients.

## Background

Intracranial hypotension (IH) is a rare clinical entity which can be caused by i.e. spontaneous CSF leakage, or following a lumbar puncture (Albes et al. 2012, Berroir et al. 2004; Brightbill et al. 2000; Christoforidis et al. 1998; Ferrante et al. 2010; Lai et al. 2007). Classically patients suffering from IH often present postural headache as main symptom and/or nausea, vomiting, or diplopia (Lin et al. 2002). Furthermore, hearing disturbances are described in about 70% of cases and are sometimes the only sign of ICH (Ferrante et al. 2010). Symptoms usually worsen when the patient is an upright position and mostly disappear when the patient lies down. In general, patients have minor symptoms, nevertheless severe neurological deterioration up to coma especially in case of misdiagnosis are described. Typical MRI findings are diffuse pachymeningeal enhancement, subdural effusion, and descent of the midbrain. Subdural hematomas (SDH) or hygromas can be concomitantly seen in 10%–69% (Lai et al. 2007, de Noronha et al. 2003). For patients with head trauma, generally a supine positioning of approximately 30° is common strategy but obviously may worsen the symptoms if IH is not suspected. Two cases of trauma-associated IH are described.

## Case description I

**Figure 1:**
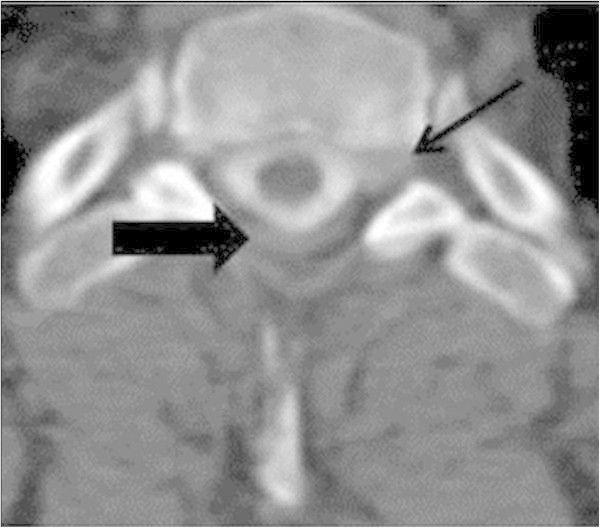
**Case I: Axial Myelo- CT presenting the point of leakage on the left side, D1 nerve (narrow 1) and the indirect leakage around the spinal cord (narrow 2).**

**Figure 2:**
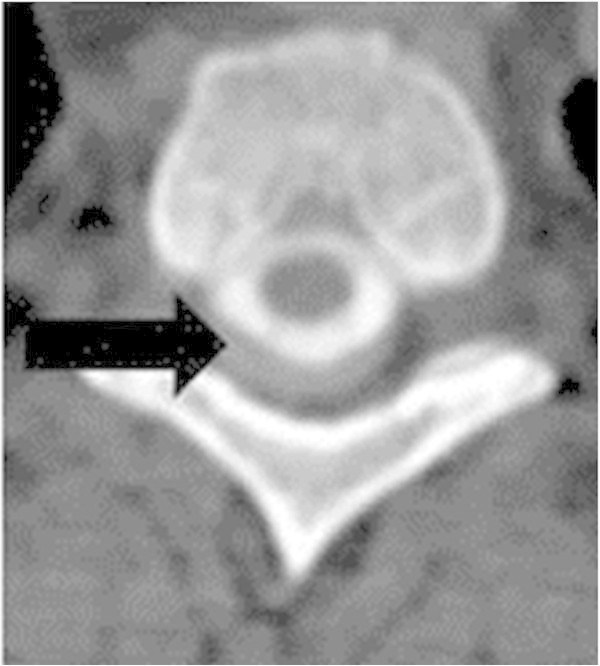
**Case I: Axial Myelo- CT demonstrating contrast leakage around the spinal cord.**

A 53-year-old previously healthy man presented with a cognitive decline, somnolence, amnestic aphasia, left-sided hemisyndrome and a history of posture-independent permanent headache, progressing over the past 10 days. A minor ski accident with a mild head injury four months ago had been reported. Clinical examination revealed a Glasgow Coma Scale (GCS) Score of 15, with discrete neurological deficits and a slowed psychomotor activity. Admission cranial magnetic resonance imaging (MRI) showed large bilateral chronic SDHs and a 7 mm midline deviation towards the left side. Two bilateral hollow bolt screws and drainage were performed. In the follow-up two revision surgeries because of acute and subacute SDHs were necessary and the patient on the neurointensive care in a supine position 30°. At that time, the patient was awake and fully recovered but six hours after the last surgery, the patient deteriorated to a GCS 4 with decerebrate posturing of his upper extremities. A computer tomography (CT) scan demonstrated sufficient evacuation of the bilateral SDH without any residual signs of mass effect. Four hours later the patient developed a rightsided mydriasis and a second CT demonstrated no change. A fourth explorative rightsided revision demonstrated no mass effect explaining the mydriasis and finally intracranial hypotension was suspected. After changing to a Trendelenburg position (-20°) the unilateral mydriasis resolved. With regards to the rapid neurological deterioration which developed within minutes, epileptic seizures activities by electroencephalogram (EEG) or a stroke were excluded. The MRI demonstrated elongation of the mesencephalon. A subsequent spinal MRI and scintigraphy showed no proof of dura fistula. A myelo-CT demonstrated a subtle leak at the dorsal roof exit zone D1 (Figures 1 and 2). An epidural blood-patch (using a mixture of 5 ml fibrin and 5 ml of glue and 5 ml blood) was prepared and 9 ml injected at that level. The epidural punction was performed under fluoroscopy, but not the mixture injection. No contrast was added to the glue/fibrin mixture, so not visible under fluoroscopy. The patient improved to a GCS 14 within hours. The patient was discharged with a GCS 15 but a lack of intellectual flexibility and difficulties in calculating and writing. After one year the patient he described his health status himself as very good.

## Case description II

**Figure 3:**
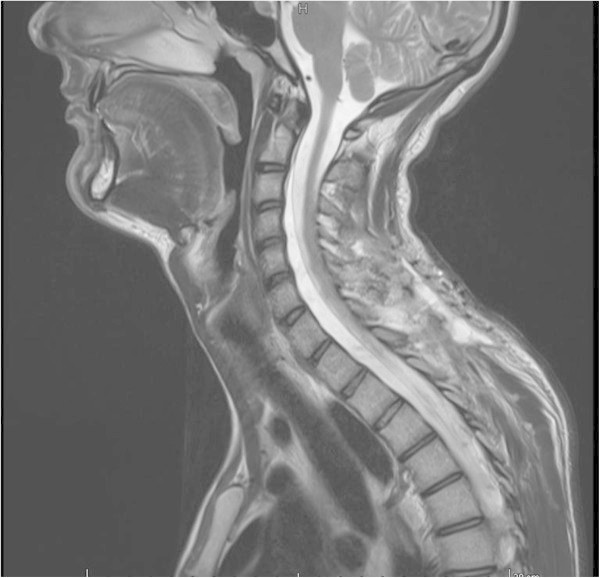
**Case II: sagittal- weighted T2 weighted MRI demonstrating a ventral subdural collection at C2- T1.**

**Figure 4:**
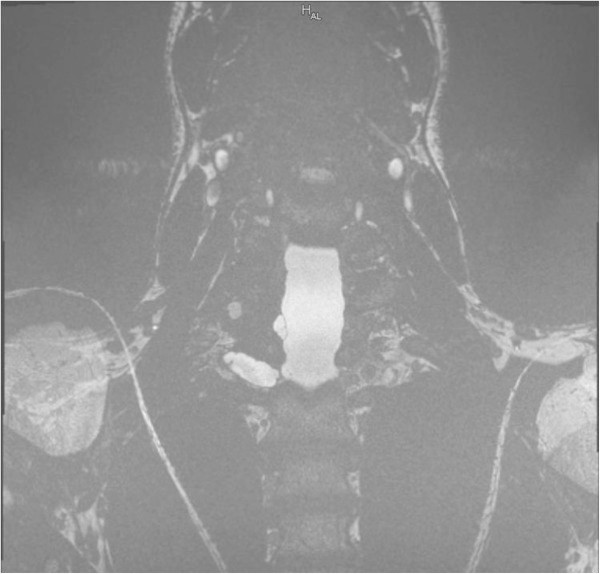
**Case II: coronar- weighted constructive interference in steady state of 6th sequence demonstrating a collection at the level of the traumatized nerve roots C7 and T1.**

**Figure 5:**
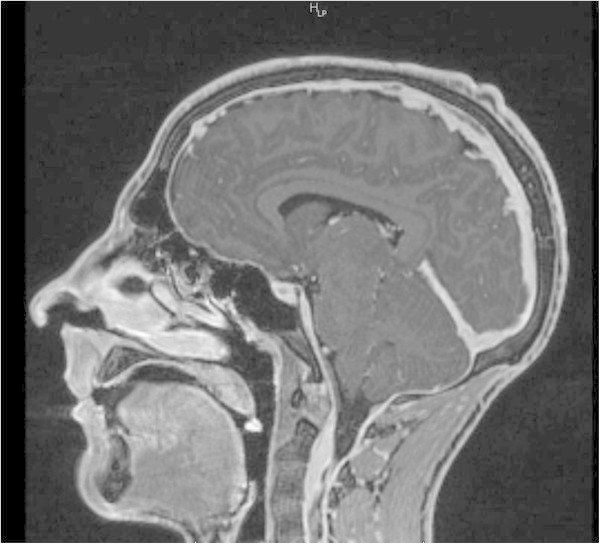
**Case II: sagittal- weighted T1 MRI with contrast medium demonstrating dural enhancement and descent of the cerebellar tonsil.**

A 20-year-old man presented in the emergency department after a motorcycle accident with paralysis of his right arm suggesting a brachial plexus lesion. He was admitted to the orthopaedic department for management of a displaced fracture of the right diaphysis of the third metatarsal bone. A spinal MRI at admission showed a ventral subdural liquid collection at C2-D1 without mass effect. Thirteen days after trauma, during a first mobilisation, he suddenly presented progressive postural headache and diplopia. Cranial MRI showed a dural enhancement and descent of the cerebral tonsils confirming IH. At the search for a leakage, a spinal control MRI was performed showing a new CSF collection at the level of the traumatized nerve roots C7 and D1. After two days resting in the Trendelenburg position the patient was operated. A right-sided fenestration of C7 to D1 was performed with an application of an epidural blood of 8 ml and fibrin glue of 8 ml. The headache resolved immediately after the intervention, and diplopia progressively declined. The patient was discharged home with favourable conditions (Figures 3, 4, 5).

## Discussion and evaluation

The German neurologist Georges Schaltenbrand described the IH the first time 1953 as “hypotension of the cerebral fluid” (Schaltenbrand 1953). The patients suffered from postural headache, bradycardia, and neck retraction symptoms, which are similar to symptoms due to an elevated intracranial pressure (ICP). Schaltenbrand himself believed that the reason for the symptoms was a state of deshydratation or even a dysfunction of the choroid plexus. Nowadays, postural headache, usually aggraveting in the upright position and improving in the supine or Trendelenburg position, is described as the cardinal symptom of IH in literature, which appears typically bilateral (Christoforidis et al. 1998; de Noronha et al. 2003). Sometimes other symptomes like nausea with vomitting or diplopia are present such in our case II (Table 1) (Berroir et al. 2004; Christoforidis et al. 1998; Ferrante et al. 2010; Lin et al. 2002; Loya et al. 2012; O’Carrol and Brant- Zawadzki 1999; Sato et al. 1998; Sencakova et al. 2001).

**Table 1:** **Review of literature listed according to the year of publication**

Reference	n	Symptoms (n =%)	Diagnosis	Treatment (n =%)	Complications	Outcome (n =%)
Christoforidis et al. 1998	4	Postural, pharmacoresistent headache (100) diplopia (75) nausea and vomiting (25) photophobia (25) neck pain (25)	Radionuclide cisternography (25), LP (50), *MRI (25)	Bed- rest for several weeks (100) caffeine 4 g/d (25)	None	Fully recovered (100)
;O’Carrol and Brant- Zawadzki 1999	4	Headache (100) nausea (50) neck stiffness (50) diplopia (25) acoustic halluzination (25)	MRI (100), LP (75), *radio cisternography (75)	Analgesics (25) EBP (100)	Subdural fluid collection (25)	No neurological symptoms after 6 months (50) still mild headache after 4 months (25) mild symptoms after 4 years (25)
Sencakova et al. 2001	25	Headache (100) Nausea	Head MRI (96), Spine MRI (48) CT myelography (64), Cisternography (56)	One or more EBP (100) after conservative treatment failed If EBP failed, surgical treatment (28)	None	No neurological symptoms after one or more EBP (72) No neurological symptoms after surgical treatment (28)
Lin et al. 2002	15	Postural headache (100) nausea (26,7) vomiting (20) dizziness (6,7) diplopia (6,7)	MRI LP*	conservative i.v. fluid EBP		Not available
de Noronha et al. 2003	4	Headache (100) diplopia (25) neck pain (25) photophobia (25) cervical pain radiating the occiput (25) neck stiffness (25)	CT (100), MRI (50), LP (50)* Angiography (25)	Symptomatical ly treated (50) EBP (25) surgical evacuation of hygroma (25)	Bilateral subdural fluid collections with confusion (50) hydrocephalus (25) ataxia and dysarthria (25)	Asymptomatic after evacuation of subdural fluid collections (75) improved gradually (25)
Berroir et al. 2004	30	Headache (100) nausea/vomitting (70) neck pain(48) hearing disturbances (14)	MRI, CT myelography Cisternography	EBP (100) Surgical treatment (3.3)		Complete cure after
						- one EBP (57)
						- two EBP (20)
						-five EBP (3.3)
						-surgery (3.3)
Lai et al. 2007	40	headache (100)	MRI, Cisternography, CT- myelography Myelography	Hydration caffeine theophylline fludrocortisone analgesics EBP (25) surgical hematoma evacuation (5)	Decrease of (7,5) abducens nerve palsy (2,5) numbness of hands (2,5) suboccipital decompression (2,5)	Asymptomatic after one year (37.5) residual headache after one year (12,5) bed-ridden and blind after three months (2.5)
Schieving et al. 2008	94	head ache	MRI, CT myelography MR myelography	Epidural blood patch (21,3) EBP and fibrin glue (31,9) EBP and surgical intervention (40,4)	None	Good outcome (80) Poor outcome (20)
Ferrante et al. 2010	42	Headache (97,6) nausea vomitting hemianopsia diplopia	CT, MRI, CT-myelography Cisternography	EBP in Trendelenburg position and acetazolamide as pre – medication	Evacuation of bilateral SDH with mass effect (5)	Complete recovery after
						-one EBP (90)
						-two (5)
						-three EBP (5)
Loya et al. 2012	3	Headache (66,6) nausea (33.3) vomiting (33.3) somnolence (33.3)	MRI, CT, CT myelography, Myelography	Trendelenburg position (100) abdominal binder (33.3) EBP (66.6)	Acute bilateral SDH (33.3) bilateral hygromas (33.3)	No neurological deficit (100)
Albes et al. 2012	26	headache (76.9)	MRI, CT, CT myelography, Myelography	EBP (96.2)		Asymptomatic (100)

*Lumbar puncture suspected IH because of the difficulty to obtain CSF. EBP: epidural blood patch; MRI magnetic resonance imaging; CT computer tomography.

The first case is especially interesting as common treatment of a relevant chronic subdural hematoma is surgery and supine position at 0°. The situation was complicated by an acute hematoma post surgery necessiting two revisions and consecutively head elevation to 30° which was well tolerated initially. We have no explanation why the patient decompensed so rapidly to coma with right sided mydriasis as he did not communicate severe headache before. But the immediate effect of the Trendelenburg position was impressive and confirmative for IH.

There are well defined diagnostic criteria for the more common spontaneous IH (Schieving et al. 2008) taking into account the unusually broad spectrum of clinical and radiological signs of IH. Schieving et al. defined the following criterion A (demonstration of a spinal CSF leak) or, if criterion A not met, criterion B (cranial MRI changes of IH) and the presence of at least one of the following: 1) low opening pressure (≤60 mmH_2_O, monitored in lateral decubitus position); 2) spinal meningeal diverticulum (mainly located in the thoracic and cervical spine); 3) improvement of symptoms after epidural blood patching. If criteria A and B not met, a criterion C is defined as the presence of all of the following or at least two of the following if typical orthostatic headaches are present: 1) low opening pressure, 2) spinal meningeal diverticulum, and 3) improvement of symptoms after epidural blood patch (Schieving et al. 2008).

In patients after brain trauma, lumbar puncture is not recommended in case of elevated intracranial pressure (Van Crevel et al. 2002; Schievink et al. 2005) and the information of a possible low lumbar opening pressure not available. Since cranial MRI is an important component for diagnosing IH, the usefulness of a CSF pressure measurement is nowadays questionable, especially in trauma patients. Nevertheless, it remains an easy-to-perform technique and adds information in spontaneous IH, as opening pressure is low in most patients (Schieving et al. 2008).

The typical MRI findings (criterion B) suggestive of IH include diffuse pachymeningeal contrast enhancement, sagging of the brain, and subdural fluid collections (Albes et al. 2012; Lin et al. 2002; Loya et al. 2012; Schievink et al. 1996). Furthermore, less specific, pituitary hyperaemia and engorgement of venous structures are described (Schieving et al. 2008). The most common and probably earliest MRI finding of IH is a diffuse, symmetrical and linear pachymeningeal enhancement as in case II though a minority of patients (28%) may have a normal MRI (Schieving et al. 2008). This pachymeningeal enhancement pattern observed in IH is specific. On the contrary, in meningitis, the enhancement localizes not only in the dura mater but also in the arachnoid and in the pia mater. After craniectomy, the pachymeningeal enhancement is tipically focal.

A further sign is the descent of the cerebellar tonsils and the elongation of the midbrain. There may be additionally cerebral or cervical subdural fluid collection, like in our patients (Figure 5).

From a pathophysiological viewpoint, IH is believed to be caused by disequilibrium of the CSF volume, usually by spontaneous CSF leaking which is typically located at higher thoracic level or cervico-thoracic junction (Mokri 2000). Two factors may provoke a spontaneous CSF leak although the exact cause is unknown: trivial trauma and dural tear (Mokri 2000; O’Carrol and Brant- Zawadzki 1999). The development of a dural tear is discussed by a genetically abnormality or deficiency of Fibrillin of Elastin. The existing vacuum is filled by expansion of the subdural blood and/or fluid. With the development of a SDH/hygroma the postural headache turns into a constant and severe headache.

IH is frequently associated with a cerebral SDH (10%–69%) as in case I (de Noronha et al. 2003). IH with subdural cervical fluid (case II) collections in a trauma context is rare (Ferrante et al. 2011) than spontaneous IH (Zakaria et al. 2013). The true mechanism of the development of SDH or hygroma due to intracranial hypotension is still unknown but there have been hypotheses proposed. First, a rupture of the bridging veins by being pulled away from the dura because of the low ICP and brain descent or secondly, a bleeding from the enlarged veins in the subdural zone may explain the development of a SDH. Hygromas are believed as compensatory enlargement of the subdural space due to the loss of CSF volume (Markwalder 1981).

Generally, it is difficult to detect the causative CSF leakage. A majority of the leakage localisation is at the higher level of the spine, particularly at the thoracic spine or at the cervico-thoracic junction (Albes et al. 2012; Mokri 2000). Recommended diagnostics to locate a CSF leakage include a cisternography/myelography with radionuclide or contrast medium if there is no evidence for a spinal CSF leak on MRI (Lai et al. 2007). In case I, a primarily performed MRI and a regular scintigraphy did not demonstrate any CSF leakage. Another scintigraphy two days later also did not present any leakage. Only a myelo-CT demonstrated a subtle leak at the cervico-thoracic junction (D1 left). In case II, a spinal MRI showed the ventral fluid collection around the traumatized nerve roots (C7-D1) suggesting a fistula at this level.

Typical treatment in the context of spontaneous IH is a non-invasive conservative approach including bed-rest in the Trendelenburg position, steroids, hydration and abdominal binder. Additionally, intravenous or oral caffeine due to the vasoconstrictive effect and a stimulation of the CSF production may be beneficial (Albes et al. 2012; Loya et al. 2012; Matsumura et al. 2000; O’Carrol and Brant- Zawadzki 1993). As alternative, an intervention is recommended, consisting of epidural blood-patches or surgical repair of the leakage as in case II (Table 1). According to the literature, the epidural blood-patch works twofold: 1) compressing the dura by volume replacement; and 2) sealing of the leakage. The latency of the effect is variable. Some patients require more than one blood patch, up to four to six. Case I received just one epidural blood-patch after which he markedly improved, nevertheless he needed a long adaptation time to a full vertical position, possibly indicating a second not identified leakage. Case II required a surgical exploration and application of blood-soaked gelfoam. Blood-soaked gelfoam, fibrin glue, or muscle, ligation or even clipping can be used to close the leak depending its position (Albes et al. 2012; Loya et al. 2012; Mokri 2000; Schievink et al. 1996).

## Conclusions

A history of mild or moderate trauma in association with prolonged postural or permanent headache can be caused by IH. Intracranial hypotension in a trauma context is rarely described and difficult to diagnose. The change from tipical supine 30° to Trendelenburg position (0–20°) can be a life-saving manoeuver in these patients.

## Consent

Written informed consent was obtained from the patient for the publication of this report and any accompanying images.
